# Hip and groin pain in physically active adults with a focus on terminology, clinical examination and imaging: A formal ESSKA‐EHPA‐ESMA Consensus

**DOI:** 10.1002/ksa.70565

**Published:** 2026-09-07

**Authors:** Per Hölmich, Adam Weir, Theodora Papadopoulou, Athanasios Papavasiliou, Kristian Thorborg, Gian Luigi Canata, Francesco Della Villa, Lior Laver, Oliver Marin‐Peña, Hannu Paajanen, Neel Badhe, Sufian S. Ahmad, Üstün Aydingöz, David Hanff, Olufemi Ayeni, Patricia Thoreux, Philippe Beaufils, The ESSKA‐EHPA‐ESMA Rating Group, Rintje Agricola, Thor Einar Andersen, Emmanuel Audenaert, Pedro Dantas, Eamonn Delahunt, Paul Dijkstra, Cristiano Eirale, Baris Kocaoglu, Thoger Persson Krogh, Bent Lund, Ajay Malviya, Vasco Mascarenhas, Jacek Mazek, Vasileios Mitrousias, Gilles Reboul, Nicolas Reina, Ernest Schilders, Reto Sutter, Zarko Vukovic, Michael Wettstein, Tobias Wörner, Vikas Khanduja

**Affiliations:** ^1^ Sports Orthopedic Research Center‐Copenhagen (SORC‐C), Department of Orthopedic Surgery Copenhagen University Hospital Amager‐Hvidovre Denmark; ^2^ Department of Orthopaedics and Sports Medicine Erasmus MC, University Medical Centre Rotterdam the Netherlands; ^3^ Sport & Exercise Medicine Sport Medicine and Exercise Clinic Haarlem (SBK) Haarlem the Netherlands; ^4^ Sport and Exercise Medicine Defence Medical Rehabilitation Centre Stanford Hall Loughborough UK; ^5^ Sports Medicine European Federation Sport Medicine Associations Lausanne Switzerland; ^6^ Department of Orthopaedic Surgery Interbalkan Medical Center of Thessaloniki Thessaloniki Greece; ^7^ Centre of Sports Traumatology and Arthroscopic Surgery Koelliker Hospital Torino Italy; ^8^ Department of Orthopaedics and Traumatology Institute of Sports Medicine Torino Italy; ^9^ Education and Research Department, Isokinetic Medical Group FIFA Medical Centre of Excellence Bologna Italy; ^10^ Department of Orthopaedics Hillel Yaffe Medical Center (HYMC) Hadera Israel; ^11^ Department of Orthopaedics, Rappaport Faculty of Medicine Technion University Hospital (Israel Institute of Technology) Haifa Israel; ^12^ Sport and Arthroscopy Unit Arthrosport Clinic Hod‐Hasharon and Tel‐Aviv Israel; ^13^ Hip Surgery Unit, Orthopaedics and Traumatology Department Hospital Universitario Infanta Leonor Madrid Spain; ^14^ Department of Surgery University of Eastern Finland Kuopio Finland; ^15^ School of Medicine University of Cambridge Cambridge UK; ^16^ Orthopaedic Department Hannover Medical School Hannover Germany; ^17^ Department of Radiology Hacettepe University School of Medicine Ankara Turkey; ^18^ Department of Radiology SSM Health Saint Louis University Hospital St. Louis Missouri USA; ^19^ Erasmus MC, Radiology and Nuclear Medicine, Erasmus University Medical Centre Rotterdam the Netherland; ^20^ Division of Orthopaedic Surgery McMaster University Hamilton Ontario Canada; ^21^ Hôpital Hôtel Dieu–Assistance Publique Hôpitaux de Paris _ Arts et Métiers Institute of Technology, EPF Engineering School, Université Sorbonne Paris Nord, IBHGC–Institut de Biomécanique Humaine Georges Charpak Paris France; ^22^ Department of Orthopaedics and Traumatology Centre Hospitalier de Versailles Le Chesnay France; ^23^ Department of Sports Medicine Oslo Sports Trauma Research Center, the Norwegian School of Sport Sciences Oslo Norway; ^24^ The Norwegian Football Association's Sports Medicine Center Oslo Norway; ^25^ Department of Orthopedic Surgery and Traumatology Ghent University Hospital Ghent Belgium; ^26^ Orthopaedic and Traumatology Center Hospital CUF Descobertas Lisbon Portugal; ^27^ NOVA Medical School Universidade Nova de Lisboa Lisbon Portugal; ^28^ School of Public Health, Physiotherapy and Sports Science, University College Dublin Dublin Ireland; ^29^ Institute for Sport and Health University College Dublin Dublin Ireland; ^30^ Aspetar, Orthopaedic and Sports Medicine Hospital Doha Qatar; ^31^ Weill Cornell Medicine Qatar Doha Qatar; ^32^ Acibadem University Faculty of Medicine Istanbul Turkey; ^33^ Department of Sports Medicine Regional Hospital of Northern Denmark Hjørring Denmark; ^34^ Molholm Private Hospital Vejle Denmark; ^35^ Department of Trauma and Orthopaedics Northumbria Healthcare NHS Trust Ashington UK; ^36^ Faculty of Medical Sciences Newcastle University Newcastle UK; ^37^ Musculoskeletal Imaging Unit Hospital da Luz Lisbon Portugal; ^38^ Faculdade de Medicina Universidade Católica Portuguesa Lisbon Portugal; ^39^ Orthopaedic and Trauma Teaching Hospital Center of Postgraduate Medical Education Otwock Warsaw Poland; ^40^ Enel‐Med Center Hospital Warsaw Poland; ^41^ Department of Anatomy Faculty of Medicine, School of Health Sciences University of Thessaly Larissa Greece; ^42^ Hernia Center, Clinique du Sport, Bordeaux‐Mérignac Mérignac France; ^43^ Department of Orthopedic Surgery and Trauma Hopital Pierre Paul Riquet, CHU Toulouse France; ^44^ Department of Orthopaedic Surgery Mayo Clinic Healthcare London UK; ^45^ Carnegie School of Sport Leeds Beckett University Leeds UK; ^46^ Department of Radiology Balgrist University Hospital Zurich Switzerland; ^47^ Faculty of Medicine University of Zurich Zurich Switzerland; ^48^ Sports Groin Pain Center VS Clinic Belgrade Serbia; ^49^ Institut de Traumatologie et d'Orthopédie du Léman Suisse Lausanne Switzerland; ^50^ Service d'Orthopédie‐Traumatologie, HRC‐Hôpital de Rennaz Rennaz Switzerland; ^51^ Faculté de Médecine et de Pharmacie Laayoune Université Ibn Zohr Agadir Morocco; ^52^ Department of Health Sciences Lund University Lund Sweden; ^53^ Department of Molecular Medicine and Surgery Stockholm Sports Trauma Research Center, Karolinska Institutet Stockholm Sweden; ^54^ Young Adult Hip Service Addenbrooke's Hospital, Cambridge University Hospitals Cambridge UK

**Keywords:** clinical examination, consensus, groin pain, hip pain, imaging, terminology

## Abstract

**Purpose:**

This European Society of Sports Traumatology, Knee Surgery and Arthroscopy–European Hip Preservation Associates–European Sports Medicine Association (ESSKA‐EHPA‐ESMA) formal consensus aims to provide evidence‐ and expert opinion‐based recommendations for the terminology, clinical examination and imaging concerning the assessment of athletes and physically active adults with hip and groin pain.

**Methods:**

The ‘ESSKA Formal Consensus’ methodology, involving a rigorous, iterative peer review process, was utilized. A multidisciplinary group of 57 experts from 22 countries formed a steering group (15 experts), rating group (20 experts) and reader group (22 experts). The steering group developed 13 questions, including four conclusive questions, across three domains: terminology, clinical examination and imaging. Statements were graded A (high‐level scientific evidence) to D (expert opinion) and scored 1–9 according to the level of agreement by the rating group. The reader group assessed statements for clarity and geographic adaptability.

**Results:**

Median ratings for the 13 statements ranged from 8 to 9 out of 9. Strong agreement was recorded for five questions, relative agreement for five questions, and three questions remained uncertain. All statements were graded C. Recommended terminology includes adductor, iliopsoas, inguinal, pubic and hip joint‐related groin pain. Clinical examination should include gait assessment, Trendelenburg's test, palpation and resistance testing of relevant muscle groups, hip range of motion and the flexion, adduction, internal rotation (FADIR) test. Imaging examinations are neither fully sensitive or specific and should always be used in conjunction with history and examination. Depending on the suspected pathology, plain radiographs or ultrasonography should be used as first‐line investigations, with magnetic resonance imaging, computed tomography and diagnostic hip injections reserved as second‐line investigations.

**Conclusion:**

This consensus provides a structured reference frame for the terminology, examination and imaging for athletes and physically active adults with hip and groin pain. Different and combined aetiologies for hip and groin pain exist, and therefore consistent terminology and systematic clinical examination are essential. Red flags must not be ignored, and imaging must be interpreted alongside history and examination findings.

**Level of Evidence:**

Level I.

**Abbreviations**
AP: anteroposterior
ASIS: anterior superior iliac spine
BKFO: bent knee fall out test
CT: computed tomography
EHPA: European Hip Preservation Association
ESMA: European Sports Medicine Association
ESSKA: European Society of Sports Traumatology, Knee Surgery and Arthroscopy
FADIR: flexion, adduction, internal rotation test
FAI: femoroacetabular impingement
FAIS: femoroacetabular impingement syndrome
HAS: Haute Autorité de Santé
MRA: magnetic resonance arthrography
MRI: magnetic resonance imaging
SMD: standardized mean difference
US: ultrasonography

## INTRODUCTION

Hip and groin pain in young and middle‐aged athletes and active adults is common and accounts for up to 10% of sports medicine consultations [14, 26, 36, 39, 49]. Pain‐related limitations can cause significant functional impairment and even cessation of an athletic career [13]. The groin, being a large and complex anatomical region, is often the focus of pain from numerous structures in the region, but also of radiating pain from outside the region. Injuries or pain from muscles and tendons constitute the majority of diagnoses in physically active adults. It is also important to recognize that patients often present with comorbid and overlapping conditions, with pain arising from multiple anatomical sources.

In recent years, there has been an increased focus on hip joint‐related causes for groin pain [34, 37]. The treatment options for hip and groin pain range from non‐surgical treatments, such as physical training and rehabilitation, local injections, manual treatment and other types of therapy, to surgical interventions, including open or laparoscopic hernia surgery, tenotomies, osteotomies or hip arthroscopy [10].

Until recently, the terminology and definitions in this field have been inconsistent, which has given rise to confusion. A review of 72 studies revealed that 33 different terms have been used to describe the diagnosis of groin pain [48]. Similarly, a Delphi consensus showed that there is significant heterogeneity of descriptions, definitions and terminology by different experts for the same clinical symptomatology [52]. This lack of consistency in terminology has given rise to a lack of a consistent method of clinical examination and consistent indications for imaging and rehabilitation in patients presenting with hip or groin pain. Since 2015, a number of specific agreement and consensus papers have been published, moving the field substantially [11, 17, 39, 52].

However, despite a growing evidence base, there remains no clear framework guiding terminology, clinical assessment or imaging in physically active adults with hip and groin pain. This lack of standardization hinders clear communication and consistent evidence‐based decision‐making. The purpose of this new consensus report was to achieve consensus on terminology, clinical examination and imaging amongst all the groups treating patients with hip and groin pain.

The aim of the European Society of Sports Traumatology, Knee Surgery and Arthroscopy (ESSKA), European Hip Preservation Associates (EHPA) and European Sports Medicine Associates (ESMA) Formal Consensus group was to build a foundation for communication in terms of terminology, clinical examination and radiological investigations that one would request when presented with a physically active adult with hip or groin pain. This framework will provide a universal language for the multidisciplinary clinicians treating this patient cohort and will facilitate better alignment in future research.

## MATERIALS AND METHODS

The consensus process followed the ESSKA methodology for a formal consensus as described by the French National Healthcare Institution Haute Autorité de Santé (HAS) [18] as well as by ESSKA [4, 5].

This approach included a group of experts in hip preservation surgery, sports medicine physicians, hernia/groin surgeons, radiologists and physiotherapists working through phases. The process involved multiple stages, including several online meetings, literature reviews, structured discussions and voting rounds (detailed methodology can be accessed in [4, 5]. Three groups were created: a steering group (15 experts) chaired by two chairpersons (V. K. and P. H.) and assisted by an ESSKA consensus project advisor (P. B.); a rating group (20 experts from 15 countries) and a reader group (22 experts from 12 countries, nominated by the ESSKA national affiliated societies). All these groups worked fully independently except for a combined steering and rating group's face‐to‐face meeting at the end of the process for final validation.

The steering group was initially divided into two. One group developed a set of questions addressing the various aspects of hip and groin pain. The questions, divided into three main sections, namely, terminology (Question 1), clinical examination (Questions 2–6) and imaging (Questions 7–13) and were prioritized based on clinical relevance and importance (Table 1). After the question preparation, the other part of the steering group performed a comprehensive literature search in order to scientifically inform the discussions and decisions as much as possible.

**Table 1:** Summary of the questions with grade of recommendation, median and range of the rating group score and related grade of agreement.

Question	Question title	Grade of recommendation	Median rating score (range)	Grade of agreement (from rating scores)
Terminology and classification				
1 (Conclusive question)	Which terminology and definitions for hip and groin pain in physically active adults are recommended?	C	9 (6–9)	Relative agreement
Clinical examination				
2	What clinical examination is relevant for determining the source of hip and groin pain?	C	8 (7–9)	Strong agreement
3	Where do patients with groin pain with known intra‐ or extra‐articular pathologies report their pain locations?	C	8.5 (6–9)	Relative agreement
4	Is there an association of clinical examination tests of the hip and groin region and imaging findings?	C	8.5 (4–9)	Uncertain agreement
5	Are clinical examination tests able to identify hip joint‐related causes of groin pain in physically active adults?	C	9 (7–9)	Strong agreement
6 (Conclusive question)	What clinical examination tests are recommended in physically active adults with hip and groin pain?	C	9 (6–9)	Relative agreement
Imaging				
7	Are imaging examinations of the hip joint able to differentiate between those with and without symptoms?	C	8 (1–9)	Uncertain agreement
8	How are imaging examinations of the hip used to identify and measure cam and pincer morphology and acetabular coverage?	C	8 (7–9)	Strong agreement
9	How are imaging examinations of the hip joint and groin region used to identify pathology?	C	9 (7–9)	Strong agreement
10	Are imaging examinations of the hip and groin able to give information about the prognosis?	C	8 (2–9)	Uncertain agreement
11	Are diagnostic injections with local anaesthetic into the hip joint able to identify intraarticular pathology?	C	9 (5–9)	Relative agreement
12 (Conclusive question)	When is imaging indicated for physically active adults with hip and groin pain?	C	9 (7–9)	Strong agreement
13 (Conclusive question)	In physically active adults with hip and groin pain, which imaging examinations are recommended as first‐line and second‐line examinations?	C	9 (6–9)	Relative agreement

Literature searches were conducted independently, using three databases: Google Scholar, PubMed and ScienceDirect. Articles were included if they were written in the English language and conducted to investigate topics relevant to this consensus. Review articles, case reports, case series, expert opinions or basic science studies were excluded. Following title and abstract screening, articles were further scrutinized through full‐text screening to generate a final list of included articles. The steering group used findings from each literature review to inform the development of a concise ‘literature summary’ for each question. For each question, a statement was proposed and assigned a grade of recommendation according to the level of supporting evidence: A (high level of scientific support), B (scientific presumption), C (low level of scientific support) or D (expert opinion) [5].

These statements were then subjected to two rounds of in‐depth anonymous rating by the rating group. During each round of rating, each statement was rated according to scientific quality and clinical experience using a Likert scale ranging from 1 (totally inappropriate) to 9 (totally appropriate). The final agreement was classified as Strong (median value ≥7, and the ratings were all ≥7) or Relative (median value ≥7, all scores ≥5) or Uncertain. A face‐to‐face combined meeting (steering+rating groups) was then conducted, enabling discussion and refining each statement collaboratively. Revised statements were then returned to the rating group for final evaluation. In accordance with formal ESSKA Consensus rules, single outlier scores were removed when <5. The final stage involved the reader group evaluating the clarity, regional adaptability and overall acceptance of the statements. A total of 22 experts from 12 affiliated societies were contacted by the ESSKA Office with a request for experts to assess the geographic adaptability of the document.

The whole document, including literature summaries and a complete list of references, is accessible on the ESSKA website: https://esskaeducation.org/esska-consensus-projects.

## RESULTS

All 13 statements in this consensus were graded C, indicating a low level of scientific evidence support and a strong reliance on clinical expertise in the development of finalized statements. Agreement across the expert rating group was consistently high, with median ratings ranging between 8 and 9 out of 9. Strong agreement was recorded for five questions, relative agreement was recorded for five questions, and three questions remained uncertain (Table 1). The conclusive questions for terminology and classification and clinical examination both achieved relative agreement with a median rating of 9 (range 6–9). The conclusive questions for imaging, when imaging is indicated, and which imaging examinations are recommended achieved strong and relative agreement, respectively, with median ratings of 9 (range 7–9) and 9 (range 6–9). No questions‐statements were removed during the process.

## QUESTIONS‐STATEMENTS

### Terminology

Which terminology and definitions for hip and groin pain in physically active adults are recommended? (Conclusive question)

A clinical entity is defined as a group of possible diagnoses, all related to a certain anatomical structure and connected by symptoms and specific clinical tests. It is important to recognize that clinical entities are not mutually exclusive, and multiple entities often co‐exist. The following list of clinical entities is not exhaustive and patients presenting with groin pain may also have symptoms arising from other sources, including other muscles, tendons, bones and nerves in the region. Additionally, pain from non‐musculoskeletal conditions and red flags should not be missed and must be considered, particularly when symptoms cannot easily be classified into one of the commonly defined clinical entities for groin pain. Clinical assessment should always consider the mechanism of injury, history and pain pattern along with the clinical findings.

The groin is defined as the junctional area between the torso and the anterior and medial thigh. The terms adductor, iliopsoas, inguinal, pubic and hip‐joint‐related groin pain should be used to describe the diagnosis of the common clinical entities of groin pain (Figure 1). For hip joint‐related groin pain, the following terminology should be used to describe the diagnosis: femoroacetabular impingement syndrome (FAIS), acetabular dysplasia and/or hip instability and conditions with or without a distinct osseous morphology such as ligamentum teres pathology, labral pathology and chondral pathology. Additionally, clinicians are recommended to use the terms cam and/or pincer ‘morphology’ when describing radiological findings underlying the diagnosis FAIS.

**Figure 1:**
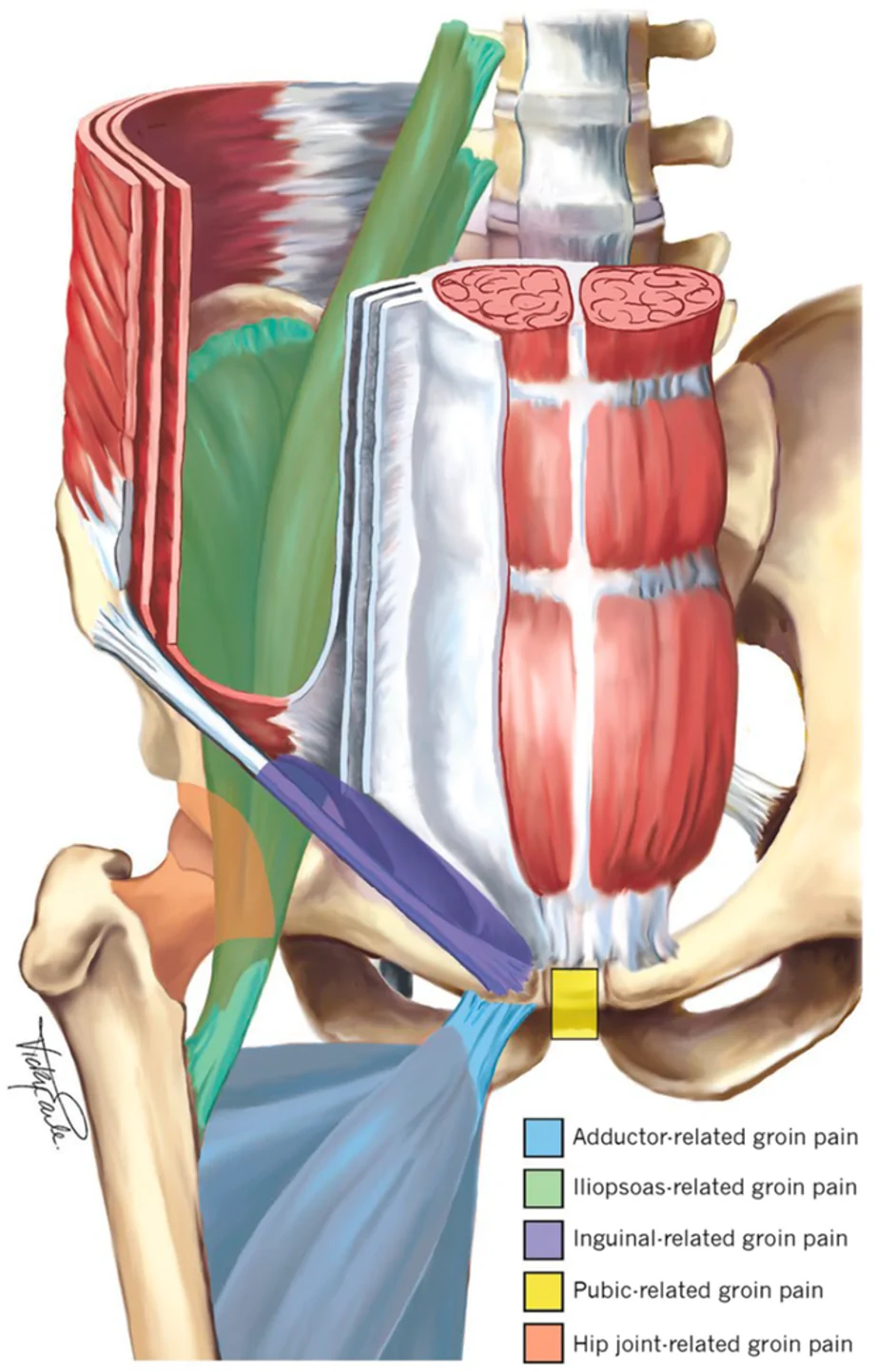
Recommended terminology for hip and groin pain in physically active adults. Figure modified from the original Doha consensus (http://dx.doi.org/10.1136/bjsports-2015-094869) by Vicky Earle.

### Grade of recommendation: C

Consensus agreement: Median 9 (range 6–9), relative agreement

A. Clinical examination

What clinical examination is relevant for determining the source of hip and groin pain?

Clinical assessment should consider the mechanism of the injury, history and pain pattern. It is important to recognize that clinical entities are not mutually exclusive, and they often co‐exist. Routine clinical examination should include palpation of the adductors, iliopsoas, inguinal canal and pubic symphysis (Figure 2) and resistance testing of hip adduction (adductors), hip flexion (rectus femoris and iliopsoas) and trunk flexion (abdominals). The FADIR (flexion, adduction and internal rotation) test and assessment of hip range of motion can rule out the hip joint pathology as a source of groin pain when painless (i.e., negative), but are non‐specific when painful (i.e., positive). Clinicians should also recognize that groin pain may originate from other regions, including the lumbosacral spine, sacroiliac joint, abdomen and pelvis. In addition, other structures around the hip, including nerves, tendons and bursae, may contribute to symptoms and should be considered during clinical examination.

**Figure 2:**
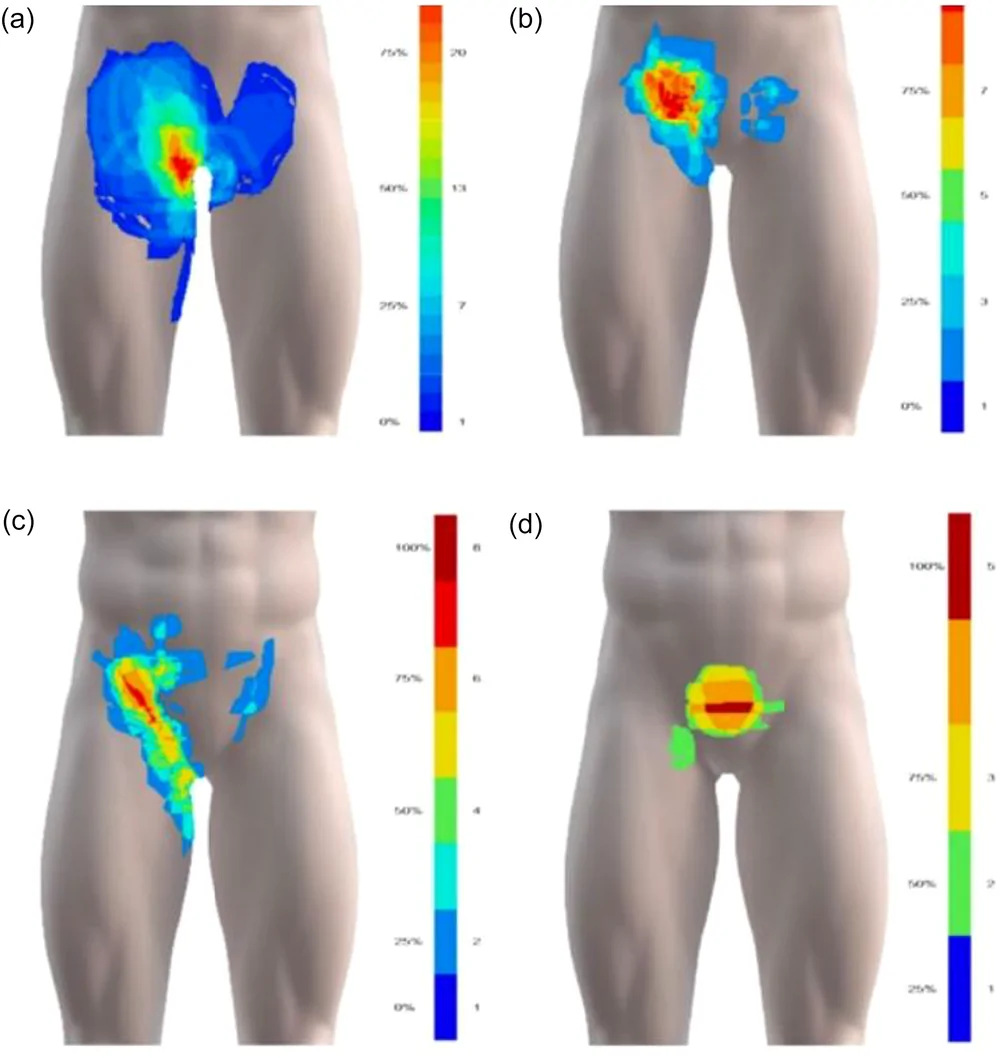
Pain map overlays of (a) adductor‐, (b) inguinal‐, (c) iliopsoas‐ and (d) pubic‐related groin pain showing common regions for each clinical entity. The colour‐scheme range reflects the maximum overlap and the minimum one overlapping area, shown in percent and the absolute number of drawings. Areas drawn by only one individual are not shown in the summary figures. Before overlaying, unilateral left‐sided pain drawings were flipped right, and bilateral pain drawings were flipped, so the largest drawn area is on the right side. This figure was reproduced from Serner et al. [47].

### Grade of recommendation: C

Consensus agreement: Median 8 (range 7–9), Strong agreement

Where do patients with groin pain with known intra‐ or extra‐articular pathology report their pain locations?

There is considerable overlap in where patients with intra‐ and extra‐articular pathologies report their pain. Clinical entities are not mutually exclusive, and clinicians must remember that patients can, and often do, have multiple clinical entities. Additionally, pain from the hip joint can also be felt in the buttock and/or lateral region of the hip. The nature of the pain does not help to differentiate between the entities.

### Grade of recommendation: C

Consensus agreement: Median 8.5 (range 6–9), relative agreement

Is there an association of clinical examination tests of the hip and groin region and imaging findings?

It is critical to remember that imaging findings often do not correlate with clinical symptoms: Positive imaging in asymptomatic, physically active individuals is common and a lack of imaging findings does not rule out a clinical diagnosis. However, both clinical assessment and imaging provide useful insights for diagnosis. A decrease in internal rotation in 90° of hip flexion and/or hip abduction is associated with a higher alpha angle, femoral neck retrotorsion and/or acetabular retroversion. Ultrasonography (US) can be useful for adductor‐related groin pain with insertion site tenderness. There is no high‐level evidence to support an association between clinical examination tests and imaging findings in relation to inguinal‐related groin pain or iliopsoas‐related groin pain.

### Grade of recommendation: C

Consensus agreement: Median 8.5 (range 4–9), uncertain agreement

Are clinical examination tests able to identify hip joint‐related causes of groin pain in physically active adults?

It is difficult to diagnose hip joint‐related groin pain based on a single clinical examination test. Patients can have intra‐ and extra‐ articular sources of groin pain, and clinical examination must be interpreted in conjunction with medical history and imaging studies and/or diagnostic injections where appropriate to make a diagnosis. If groin pain is elicited during the FADIR test or during testing of the hip range of motion, the hip may be causing the groin pain. However, if there is no reproduction of groin pain, the hip joint is unlikely to be the source of pain. Additionally, if muscle resistance testing and palpation reproduce groin pain, extra‐articular pathology is a likely source of groin pain and should be suspected.

### Grade of recommendation: C

Consensus agreement: Median 9 (range 7–9), Strong agreement.

What clinical examination tests are recommended in physically active adults with hip and groin pain? (Conclusive question)

Clinical examination of patients with hip and groin pain needs to be interpreted in conjunction with medical history and imaging studies and/or diagnostic injections where appropriate to make a diagnosis. It is common for a single patient to have pain arising from multiple clinical entities. A systematic clinical examination of the hip and groin should be performed in all cases, including gait assessment, Trendelenburg test, palpation and resistance testing of the adductors, iliopsoas, inguinal canal and pubic symphysis, with assessment of the hip range of motion and FADIR test. Extra‐articular pathology is likely when groin pain is elicited by resistance testing and muscle palpation, whereas intra‐articular pathology is more likely when pain is elicited during hip range of motion assessment or the FADIR test, but unlikely when these tests are painless. No test for hip instability has a high specificity. Rotational and/or version abnormalities of the femur and/or tibia should be considered; therefore, assessment of hip range of movement while prone should be performed. When young and active individuals present with groin pain, it is important not to neglect the possibility of serious pathology.

### Grade of recommendation: C

Consensus agreement: Median 9 (range 6–9), relative agreement

B. Imaging

Are imaging examinations of the hip joint able to differentiate between those with and without symptoms?

Imaging alone cannot differentiate between those with or without symptoms. The relationship between intra‐articular hip joint morphology or pathology on imaging and the presence of groin pain remains uncertain, with cam morphology frequently visible on plain radiographs in asymptomatic individuals. Pain is associated with cam and mixed FAI morphologies, articular cartilage pathology, ligamentum teres tears and bone marrow oedema, but not with pincer morphology. Imaging studies are usually static, which does not reflect the dynamic nature of hip impingement and instability, and hence, is a major limitation of imaging. Routine screening of the hip joint in asymptomatic individuals is not warranted.

### Grade of recommendation: C

Consensus agreement: Median 8 (range 1–9), uncertain agreement

How are imaging examinations of the hip used to identify and measure cam and pincer morphology and acetabular coverage?

Imaging examinations quantitatively assess the hip joint morphology and can identify cam or pincer morphology, acetabular dysplasia and the femoral and acetabular rotational profile. Cam morphology can be assessed using an anteroposterior (AP) radiograph and Dunn view (or similar lateral view), computed tomography (CT) or radial MRI. Pincer morphology can also be assessed using AP radiographs, with the lateral centre‐edge angle being the most reliable sign. Acetabular dysplasia can be evaluated on AP radiographs using measurements such as the lateral centre‐edge angle and acetabular index. Rotational profile of the femur and acetabulum can be assessed through measurement of femoral and acetabular version using CT or MRI.

### Grade of recommendation: C

Consensus agreement: Median 8 (range 7–9), strong agreement.

How are imaging examinations of the hip joint and groin region used to identify pathology?

Imaging can identify chondrolabral pathology; however, the absence of radiographic evidence of osteoarthritis does not exclude chondrolabral pathology. Magnetic resonance imaging (MRI) is preferred to CT for the evaluation of chondrolabral pathology, and where possible, 3 Tesla or higher is preferred to 1.5 Tesla as it is more sensitive and equally reliable. There are no optimal classification systems for labral lesions, and ideally, imaging reports should be descriptive. Bone marrow oedema and adductor tendon changes are common findings in asymptomatic athletes and of uncertain significance. Radiographs and MRI can assess the maturity of the pubic symphysis to assess for pubic apophysitis. There is a high risk of false‐positive findings on dynamic US for inguinal‐related groin pain. There is no clear role for imaging in iliopsoas‐related groin pain. The weak associations and high prevalence of findings in asymptomatic individuals emphasize the need for imaging to be interpreted alongside the clinical findings.

### Grade of recommendation: C

Consensus agreement: Median 9 (range 7–9), strong agreement.

Are imaging examinations of the hip and groin able to give information about the prognosis?

There are very few studies that have investigated the role of imaging in the prognosis of hip and groin pain in physically active adults. Consequently, the exact role of imaging for prognosis remains unclear.

### Grade of recommendation: C

Consensus agreement: Median 8 (range 2–9), uncertain agreement.

Are diagnostic injections with local anaesthetic into the hip joint able to identify intraarticular pathology?

Pain relief obtained from a local anaesthetic intra‐articular hip injection can reliably differentiate intra‐articular pathology from extra‐articular pathology. The local anaesthetic intra‐articular hip injection should be guided by either US or fluoroscopy. Local anaesthetic intra‐articular hip injection should be used in addition to history, examination, imaging and other investigations to make a diagnosis.

### Grade of recommendation: C

Consensus agreement: Median 9 (range 5–9), relative agreement.

When is imaging indicated for physically active adults with hip and groin pain? (Conclusive question)

Findings on imaging do not necessarily correlate with hip and groin symptoms in physically active adults. Consequently, imaging should only be performed in symptomatic patients following a systematic clinical assessment. It is important to recognize that symptomatic patients may have normal imaging, and asymptomatic patients may have abnormal imaging. Current evidence does not support routine imaging screening for asymptomatic individuals. Imaging provides morphometric analyses of the hip and pubic symphyseal joint and depicts the surrounding soft tissues. Imaging should be considered in cases with clinical suspicion of underlying pathology, in patients with hip joint‐related pain that has not improved after a short course of non‐surgical treatment, and in patients with groin pain unresponsive to non‐surgical treatment when the clinical diagnosis remains uncertain.

### Grade of recommendation: C

Consensus agreement: Median 9 (range 7–9), Strong agreement.

In physically active adults with hip and groin pain, which imaging examinations are recommended as first‐line and second‐line examinations? (Conclusive question)

The cost of imaging, including financial resources, workforce demands and time, in addition to the burden of ionizing radiation exposure, must be considered before performing investigations. The first‐line imaging examination for the morphological evaluation of the hip and groin is an AP pelvic radiograph with a Dunn view or similar lateral view. US is the first‐line imaging examination for an inguinal hernia and for adductor‐, inguinal‐ or iliopsoas‐related groin pain. MRI, CT and diagnostic hip injections are second‐line investigations. MRI or magnetic resonance arthrography (MRA) should be used for assessment of FAIS and acetabular dysplasia, including evaluation of femoral torsion with axial images through the distal femur, assessing poor prognostic factors for hip preservation surgery, including joint space narrowing, femoral or acetabular cysts and acetabular bone oedema, and for evaluation of pubic‐, iliopsoas‐ and adductor‐related groin pain. CT should be used with caution due to significant ionizing radiation exposure, unless low‐dose protocols are available. A diagnostic, image‐guided local anaesthetic intra‐articular hip injection can be used as a second‐line investigation for intra‐articular pathology.

### Grade of recommendation: C

Consensus agreement: Median 9 (range 6–9), relative agreement.

## DISCUSSION

The key message of the ESSKA‐EHPA‐ESMA consensus is that hip and groin pain frequently arises from multiple coexisting anatomical sources and cannot be reliably attributed to a single structure based on isolated clinical tests or imaging findings. Instead, accurate diagnosis requires integrating patient history, a structured and systematic physical examination and appropriate imaging interpreted within the clinical context.

The consensus statements consistently achieved high agreement scores, with strong or relative agreement for over 75% of questions, including all four conclusive questions, based on ratings from experts across disciplines and countries. This consensus indicates that despite limited robust scientific literature on the assessment of physically active adults with hip or groin pain, structured expert collaboration could yield clinically applicable and widely accepted recommendations with strong geographical adaptability and applicability.

### Terminology and classification

The need for universal terminology and taxonomy in the assessment of hip and groin pain in athletes and physically active adults was highlighted by a study conducted by Weir et al. in 2015, who found that 23 international experts used 22 different terms for a single clinical condition, prompting the development of the Doha Agreement [52]. This was a major step forward and has since become the most widely adopted framework, with usage rates between 57% and 69% among clinicians [22].

The ESSKA‐EHPA‐ESMA consensus builds on this foundation, categorizing hip and groin pain in athletes and physically active adults into five clinical entities: adductor‐related, iliopsoas‐related, inguinal‐related, pubic‐related and intra‐articular hip‐related pain. This taxonomy not only facilitates clearer communication between clinicians and patients, but also enhances research consistency and comparability, ultimately supporting more accurate diagnosis and appropriate clinical management.

### Clinical examination

Each clinical entity presents with distinct findings on structured clinical examination.

Adductor‐related groin pain is reliably identified by tenderness at the proximal adductor insertion and pain on resisted adduction, with studies demonstrating strong intra‐observer and inter‐observer agreement with this approach (*κ* = 0.92 and 0.94) [24].

Iliopsoas‐related groin pain is characterized by tenderness on palpation of the iliopsoas, which can be reliably palpated at two sites: the triangle between the inguinal ligament, the medial edge of sartorius and the femoral artery and at the level of the anterior superior iliac spine (ASIS) lateral to the rectus abdominis in the lower abdomen [50, 51]. Additionally, the elicitation of pain on resisted hip flexion or passive stretching further supports the clinical diagnosis of iliopsoas‐related pain [23].

Inguinal‐related groin pain is characterized by pain in the inguinal canal on scrotal invagination. This was found in 94% of athletes with inguinal‐related groin pain in a recent study investigating the inter‐examiner reliability of clinical examination tests for inguinal‐related groin pain in male athletes [20]. Additionally, it was found that in 20% of cases, recognizable injury pain can only be identified by scrotal invagination. It is therefore critical to conduct this as part of routine examinations. Inguinal‐related groin pain is also more likely when pain is exacerbated during resisted abdominal muscle testing, Valsalva manoeuvre, coughing or sneezing [20, 46].

Pain on palpation of the pubic symphysis and adjacent bone reliably elicits characteristic pubic‐related groin pain [21]. While no specific resistance test exists for pubic‐related groin pain, resisted adduction, which stresses the pubic symphyseal joint, can aid diagnosis.

Hip range of motion and FADIR testing are critical components of the clinical examination for identifying hip joint‐related causes of groin pain. Additionally, the bent knee fall out (BKFO), a validated combined movement test involving hip flexion, abduction and external rotation, has been shown to differentiate athletes with and without groin pain, with higher scores representing reduced range of motion (standardized mean difference [SMD] = 0.75, 95% confidence interval [CI]: 0.24–1.27) [30, 32].

A systematic review by Reiman et al. found that FADIR, also known as the anterior impingement test when conducted with 90° of hip flexion, had high sensitivity but poor specificity for both FAIS and labral tear identification [40, 53]. FADIR's high sensitivity makes it useful for ruling out the hip as a possible source of pain, but its low specificity means that a positive test can only indicate the need for further investigation [30, 41, 42, 52].

### Imaging

Imaging is a valuable tool for the assessment of physically active adults presenting with hip or groin pain. However, the high prevalence of symptomatic patients with normal imaging and asymptomatic individuals with positive imaging findings necessitates judicious use and cautious interpretation [1, 15, 19, 38]

Radiographs remain the first‐line imaging modality for assessing hip morphology, with reported sensitivities between 71% and 96% for detecting cam morphology [3, 12, 31, 45, 54].

If the suspected diagnosis is that of an inguinal hernia, the first‐line imaging examination is US. Regarding intra‐articular pathology, US shows reasonable diagnostic accuracy for labral pathology, with a reported sensitivity of 69% and specificity of 82% [16], its clinical role for the assessment of intra‐articular pathology outside injection guidance remains limited due to its inability to evaluate the entire labrum [25].

MRI and CT imaging serve as second‐line modalities. MRI and MRA are commonly used techniques for intra‐articular pathology. In a meta‐analysis conducted by Saeid et al., MRA was shown to have superior sensitivity to MRI for the detection of labral tears (91% vs. 87%) but inferior specificity (58% vs. 83%) [44]. Additionally, this study found that sensitivity was similar (75% vs. 76%) and specificity was greater with MRA than MRI (79% vs. 72%) for detection of chondral lesions. CT imaging techniques have also demonstrated high sensitivity for the detection of a wide range of pathologies [29], though its use is limited due to the risk associated with ionizing radiation exposure [9, 18, 28].

Regarding imaging prognosis, it has been well documented that cam morphology and acetabular dysplasia are associated with an increased risk of hip osteoarthritis [8]. Generally, however, there is insufficient evidence to suggest that imaging enhances prognostic assessment in physically active adults with hip or groin pain [6, 33].

While intra‐articular injections are widely accepted to be able to differentiate intra‐articular from extra‐articular causes of pain [7], its implications for prognosis remain unclear. A recent study by Pepic et al. reported that injection response does not reliably predict 2‐year postoperative outcomes [35], consistent with earlier work by Ayeni et al. and Krych et al. [2, 27]. This emphasizes the point that the decision to offer surgery requires a holistic clinical assessment and should not be solely based on intra‐articular injection response alone.

This consensus demonstrates that the current body of evidence regarding hip and groin pain assessment is currently limited in both quality and consistency, meaning that clinical expertise had to be favoured in the development of the final consensus statements.

A consensus applies similar principles to those applied in evidence‐based medicine (EBM) [43], integrating scientific evidence with clinical expertise, and its value is even more relevant when the scientific evidence is not very strong.

One limitation of the current consensus is that it provides recommendations and statements based on current knowledge (both scientific and clinical expertise) at a specific time point. However, the authors realize that knowledge in this field is likely to evolve over time, and the terminology, recommendations and statements formed in the current consensus may thus evolve and develop accordingly.

However, this consensus has several strengths. It represents an extensive, international, multidisciplinary collaboration grounded in six key principles: pluralism, iterative development, independent refereeing, structured literature review, final discussion after an anonymous process and transparency [5]. The consensus involved 57 experts across 22 countries and several disciplines, ensuring both geographical and multidisciplinary adaptability and applicability. Importantly, the project was undertaken in partnership with established professional and scientific societies (ESSKA‐EHPA‐ESMA), following the European Society of Sports Medicine, Knee Surgery and Arthroscopy (ESSKA) ‘Formal Consensus’ methodology, ensuring neutrality and scientific credibility of the recommendations.

## CONCLUSION

This consensus highlights the heterogeneity of the literature on hip and groin pain in athletes and physically active adults while providing a structured framework for the terminology, examination and imaging for clinicians. This consensus also emphasizes that different aetiologies for groin and hip pain exist, and that red flags must not be ignored. Systematic clinical examination is therefore necessary, and imaging examinations must be used in combination with history and physical examination. This will enable a universal language to be used across the multidisciplinary team, strengthening outcome consistency within this patient cohort and facilitating future research.

## AUTHOR CONTRIBUTIONS

Per Hölmich and Vikas Khanduja contributed to idea generation and provided overall supervision and responsibility for the project. All authors contributed to the investigation and methodology, participated in the review and editing processes and approved the final manuscript.

## CONFLICT OF INTEREST STATEMENT

Philippe Beaufils serves as a Scientific Advisor for the ESSKA consensus methodology. Vikas Khanduja was the ESSKA‐EHPA Chair at the time of the Consensus being developed. Patricia Thoreux was the ESSKA–ESMA Chair at the time of the Consensus being developed.

## Data Availability

The data that support the findings of this study are available from the corresponding author upon reasonable request.
